# Notes for Contributors

**DOI:** 10.1155/S1463924680000569

**Published:** 1980


estimated as 7.5 hours. The amplitude a (defined as half of the difference between the deepest and highest parts), was 5 mm. The distance of the two parallel contour lines of the noisy trace was 3 mm. The distance between the contour lines corresponds approximately to 5s, which gives a value for s of 0.6 mm.

The variance of the migration is given by:

$$\sigma^2 = \frac{a^2}{2} = \frac{5^2}{2} = 12.5$$

and

$$\sigma = 3.54 \text{ (mm)}$$

The shortest time interval between the two successive settings (if the time of the setting can be neglected) is therefore given by:

$$t_{st} = 0.29\frac{0.6}{3.54}\ 450 = 22 \text{ (min)}$$

Practically, this means that after each chromatographic separation the zero point must be reset. This observation fully agrees with the author's experience.

Thus to calculate the frequency of standardisation, the amplitude (a) and the periodic time (T) of the zero point migration from data of long term experiments must be determined. From the amplitude the standard deviation ($\sigma$) is then calculated using equation (2). If the precision (s) of the single measurements, and $\sigma$, T, and $\tau$ are known, the necessary time interval $t_{st}$ can be calculated using equation (4) or (5).

### Conclusion

The principle of the method of the calculation can be used also in those cases where the 'drift' or the change of the systematic error does not originate from the instability of an instrument, but from other sources. The change, however, must be periodic.

REFERENCES

[1] McFarren, E. F., Lishka, R. I. and Parker, J. H., Analytical Chemistry, 1970, **42**, 358.

[2] Midgley, D., Analytical Chemistry, 1977, **49**, 510.

[3] Gegus, E., Paper at the 10. Spektrometertagung, den Hagg 1974.

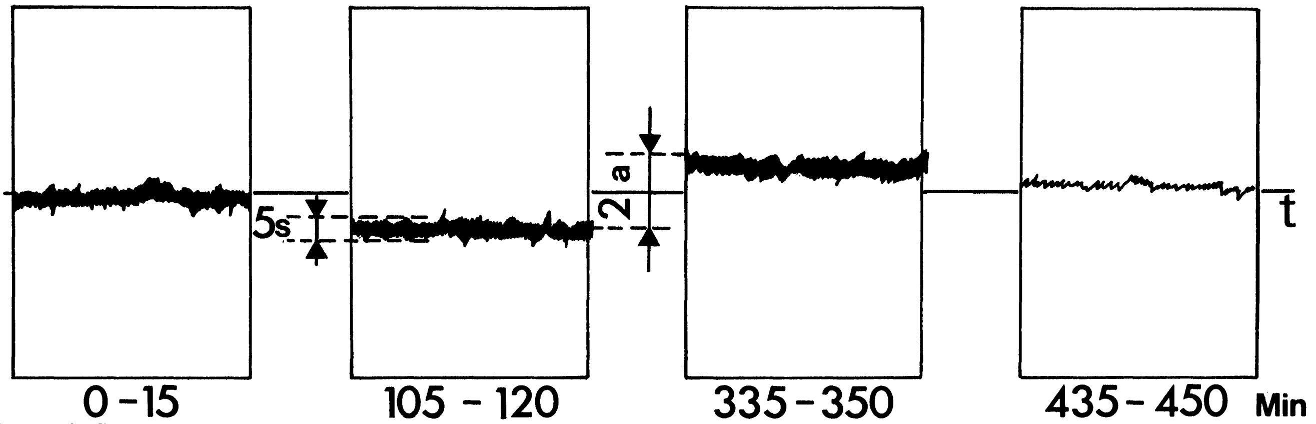

*Figure 3. Segments of an original record obtained by a liquid chromatograph. Evaluation of the parameters used for calculation.*

### Notes for Contributors

### Presentation of manuscripts

Manuscripts should be typed (double-spaced) on one side of the paper only and with generous margins. The title should be brief and informative avoiding the word "new" and its synonyms. The full list of authors with their affiliations and full address(es) should appear on the title page. On a separate sheet an abstract of no more than 150 words is required. This should succinctly describe the scope of the contribution and highlight significant findings or innovations. It should be written in a style which can easily be translated into French and German.

*The Concise Oxford Dictionary* and Fowler's *Modern English Usage* (both published by Oxford University Press) should be used as the standard for spelling and grammar. Abbreviations should be limited to those generally recognised, or where a frequently occuring term is abbreviated it should, in the first instance, be explained thus "flow injection analysis (FIA) ..." and the abbreviation used thereafter. Abbreviations, for standard measures and units should follow SI recommendations. There are various publications giving guidance on the use of SI units.

References should be indicated in the text by numerals following the author's name, i.e. Skeggs [6]. On a separate sheet of paper, list all references in numerical order thus:

[6] Skeggs, L.T., *American Journal of Clinical Pathology*, 1959, **28**, 311.

Note that journal titles are given in full. Where there is more than one author, the form Foreman et al. should be used in the text but all authors should be named in the list of references. When reference is made to a chapter in a book the reference should take the following form:

[7] Malmstadt, H.V. in "Topics in Automatic Chemistry" Ed. Stockwell P.B. and Foreman J.K. 1978 Horwood, Chichester, pp. 68-70.

Only work which has been published or has been accepted for publication should be cited. Avoid the citation of documents which are subject to restricted circulation, patent literature, unpublished work and personal communications. The latter can be mentioned in the text in parenthesis.

To illustrate a paper line diagrams are preferred to photographs. Photographs should only be used when they significantly add to the discussion. Diagrams, charts and graphs should be carefully drawn in black ink on stout card or heavy quality tracing paper. Most illustrations are reduced for publication; to allow for this originals should be between 16 and 36 cm wide (the depth must not exceed 50 cm). The lettering of diagrams should be sufficiently clear to withstand reduction. Except in the case of proper names, all lettering should be in lower case print. If photographs are used they must be supplied in the form of clear, unmounted, glossy, black and white prints. "Instant" photographs are not normally acceptable. All illustrations must be identified on the reverse showing the figure number and the author's name.

Each illustration should have a fully explanitory caption. Captions should be typed together on a separate sheet of paper; they must *not* be an inseparable part of the illustration.

